# Central Nervous System Infections Caused by Bacillus Calmette–Guerin: Case Report and Narrative Literature Review

**DOI:** 10.3390/microorganisms13061283

**Published:** 2025-05-30

**Authors:** Davide Chemello, Maddalena Albertini, Johanna Chester, Sara Esperti, Elena Ghidoni, Gabriella Orlando, Giacomo Franceschi, Corrado Iaccarino, Lucio Lucchesi, Giacomo Pavesi, Cristina Mussini, Erica Franceschini

**Affiliations:** 1 Department of Infectious Diseases, University Hospital of Modena, University of Modena and Reggio Emilia, 41125 Modena, Italy; maddalena95.albertini@gmail.com (M.A.); sara.esperti@gmail.com (S.E.); elena.ghidoni92@gmail.com (E.G.); orlando.gabriella@policlinico.mo.it (G.O.); frangia91@hotmail.it (G.F.); cristina.mussini@unimore.it (C.M.); 2 Department of Surgery, Medicine, Dental Medicine and Morphological Sciences, University of Modena and Reggio Emilia, 41125 Modena, Italy; johannachester@gmail.com; 3 School of Neurosurgery, Department of Biomedical, Metabolic and Neural Sciences, University of Modena and Reggio Emilia, 41125 Modena, Italy; corrado.iaccarino@unimore.it (C.I.); lucio.lucchesi@icloud.com (L.L.); giacomo.pavesi@unimore.it (G.P.); 4 Department of Neurosurgery, Hospital of Baggiovara, University Hospital of Modena, 41126 Modena, Italy; 5 Neurosurgery Unit, Azienda Unita Sanitaria Locale di Reggio Emilia (AUSL RE) IRCCS, 42122 Reggio Emilia, Italy

**Keywords:** *Bacillus Calmette–Guerin*, ventriculitis, encephalitis, *M. bovis*

## Abstract

*Bacillus Calmette–Guerin* (BCG) central nervous system (CNS) infections are one of the rarest complications following BCG exposure. A 77-year-old male, with bladder cancer previously treated with BCG instillation, presented with fever, confusion, and brain magnetic resonance imaging (MRI) consistent with encephalitis one month after the last BCG instillation. Cerebrospinal fluid (CSF) showed marked hypoglycorrhachia, hyperproteinorrachia, and lymphocytic pleocytosis. Despite CSF culture negativity, the presentation was considered suggestive of BCG-related encephalitis, and the empirical standard antitubercular treatment (rifampin, isoniazid and ethambutol), plus dexamethasone, was initiated. Following initial improvement, gait ataxia and hemiplegia were observed at the 4-month follow-up. MRI revealed an excluded enlarged left lateral ventricle with signs of ventriculitis, requiring surgical drainage. CSF collected during neurosurgery resulted positive on PCR for *M. tuberculosis* complex. Adjunctive linezolid was initiated, replaced by levofloxacin due to adverse events after 2 weeks. The patient was discharged following a normal CSF analysis. Oral antitubercular therapy was prescribed for 14 months and there were no signs of relapse at the 24-month follow-up. Previously, 16 cases of CNS BCGitis have been reported, without any cases of clinical relapse during antitubercular treatment. Furthermore, our study reports the use of linezolid as a 4th antitubercular drug for CNS BCGitis.

## 1. Introduction

Immunotherapy with *Mycobacterium bovis* and *Bacillus Calmette–Guerin* (BCG) is recommended by guidelines as an adjuvant intravesical treatment for non-muscle-invasive bladder cancer (NMIBC) to reduce the incidence of recurrence following the endoscopic removal of NMIBC [1,2,3,4]. Despite data suggesting that BCG intravesical immunotherapy is effective in the prevention of local NMIBC recurrence, side effects, mostly mild and self-resolving, are frequently reported [4].

The cumulative incidence of post-intravesical BCG-disseminated infections ranges from 1% to 4.8% [4,5,6,7,8,9]. The pattern of organ involvement varies, with pulmonary infections being the most common (25–50%) [4,5,6], followed by osteoarticular infections (19.9%), mycotic aneurysms (5.7%), and granulomatous hepatitis (5.7%) [4,5,6]. The median time from the last BCG instillation and the onset of symptoms is reported to be 13 days, with systemic infections generally presenting earlier than genitourinary infections [4].

In literature, disseminated BCG infections are heterogeneously defined; a large literature review published in 2014 defined disseminated BCG infections as “mycobacterial infection occurring beyond the genitourinary system” in patients who received one or more BCG instillations, responded to antitubercular therapy and without any other plausible clinical explanation [4]. However, there is no consensus on whether pathogen isolation from the affected site is necessary to define a BCG infection [4,5,6].

As CNS infections following BCG intravesical immunotherapy are rare, estimated at 0.4%, only a few case reports are available [4,10,11,12,13,14,15,16,17,18,19,20,21]. Diagnostic and therapeutic guidelines are missing.

We report a case of microbiologically confirmed BCG ventriculitis following BCG intravesical immunotherapy, as well as infection relapse despite antitubercular treatment. We include a current literature review of CNS infections associated with BCG intravesical immunotherapy or vaccination.

## 2. Case Presentation

### 2.1. Past Medical History and Presentation of Symptoms

We present the case of a 77-year-old male diagnosed with a BCG encephalitis/ventriculitis. In 2017, he was diagnosed with prostate adenocarcinoma (Gleason score 3 + 3), which was managed with ablative radiotherapy, completed in November 2017. No prostate cancer recurrence has been observed. In 2020, following the onset of hematuria, the patient was diagnosed with high-grade bladder cancer. A transurethral resection of the bladder was subsequently performed, followed by intravesical BCG immunotherapy, consisting of two cycles of induction (6 + 6 instillations) and one cycle of maintenance (3 instillations). The most recent invasive procedure was a cystoscopy, performed during an oncological bladder cancer follow-up 5 months before the patient presented with clinical symptoms of infection.

Thirty-three days after the final instillation of BCG immunotherapy, a persistent low-grade fever, a transient state of confusion, asthenia, headache, and weight loss (8 kg in a month) were observed. His general practitioner prescribed amoxicillin/clavulanate 875/125 mg twice daily per os for 7 days, but symptoms persisted despite therapy. An altered mental status emerged and the patient was admitted to the local hospital.

### 2.2. First Hospital Admission

Upon admission, the patient was tachycardic, asthenic, and febrile (38 °C). No meningeal signs were present. Blood chemistry tests were within normal ranges, with the exception of hyponatremia (134 mEq/L; reference range: 136–146 mEq/L). Additional blood and urine cultures, chest X-ray, QuantiFERON, and HIV and syphilis tests all resulted negative. IgG for *B. burgdorferi* resulted positive (35 UA/mL; reference range: >15 positive) with negative IgM (<0.9 index; reference range: >1.1 positive), confirmed with a Western blot assay (positivity in VIsE, Osp17, and p39).

During the hospital stay, his mental status further deteriorated. A lumbar puncture (LP) was performed, and cerebrospinal fluid (CSF) analysis showed lymphocytic pleocytosis (leucocytes 237 cell/mm^3^, mainly lymphocytes; reference range: >4 cell/mcL abnormal), hypoglycorrhachia (17 mg/dL; reference range: 40–80 mg/dL), and hyperproteinorrachia (250 mg/dL; reference range: 20–50 mg/dL). An empirical treatment with acyclovir ev 10 mg/kg three times a day, ampicillin ev 3g four times a day, and ceftriaxone ev 2 g two times a day was initiated due to suspicion of meningoencephalitis. The patient was transferred to our center for specialized management.

**Figure 1:**
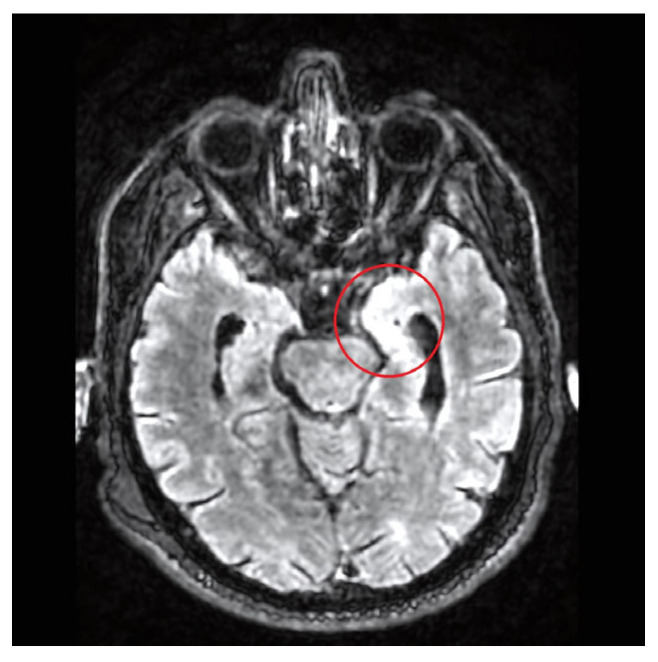
First MRI in FLAIR sequence showing vasogenic edema of the temporal lobe.

Brain magnetic resonance imaging (MRI) was prescribed, and imaging showed vasogenic edema in the hippocampus and temporal lobe regions, consistent with encephalitis (Figure 1). Electroencephalography (EEG) showed diffuse slow basal activity with no epileptiform anomaly.

CSF microbiological tests (multiplex PCR, bacterial and mycobacterial cultures) all resulted negative. A Western blot assay for *B. burgdorferi*, performed on CSF, confirmed IgG positivity (11.0 AU/mL; reference range: >5.5 positive). Acyclovir and ampicillin were discontinued. Due to suspicion of neuroborreliosis, ceftriaxone was maintained for a total of 14 days. No clinical improvement was shown during therapy.

Tumor and autoimmune markers were tested on plasma and CSF to rule out paraneoplastic encephalitis; results were negative. A total-body computed tomography (CT) scan and positron emission tomography did not show any signs of active neoplasia.

Due to persistently altered mental status, an LP was repeated, and CSF showed persistent hypoglycorrhachia, lymphocytic pleocytosis and hyperproteinorrachia. Urine culture was positive for *M. bovis* BCG strain, while mycobacterial cultures on CSF, blood and feces resulted negative.

During his hospital stay, the patient developed severe hyponatremia (Na+ 124 mEq/L). Liquid restriction was initiated, under the suspicion of syndrome of inappropriate antidiuretic hormone secretion (SIADH), but it was not sufficient in correcting the hyponatremia, and oral tolvaptan was started.

Despite the negativity of CSF mycobacterial microscopy, PCR and cultures, the persistent hypoglycorrhachia, the lymphocytic pleocytosis, and the patient’s past medical history were considered suggestive of BCG-related encephalitis. Furthermore, SIADH is a condition commonly associated with tubercular meningitis/encephalitis [22]. An empirical treatment with rifampin 600 mg/day, ethambutol 1500 mg/day, and isoniazid 300 mg/day, plus dexamethasone 32 mg/day, was started. The clinical status of the patient improved abruptly after therapy initiation. A second MRI showed reduced inflammation on the temporal lobe and EEG findings improved.

The patient was discharged and instructed to continue antitubercular therapy for 12 months, with steroid treatment to be tapered over an 8-week course. At the scheduled 2-week follow-up visit, the patient was in a generally good clinical state.

### 2.3. Second Hospital Admission

**Figure 2:**
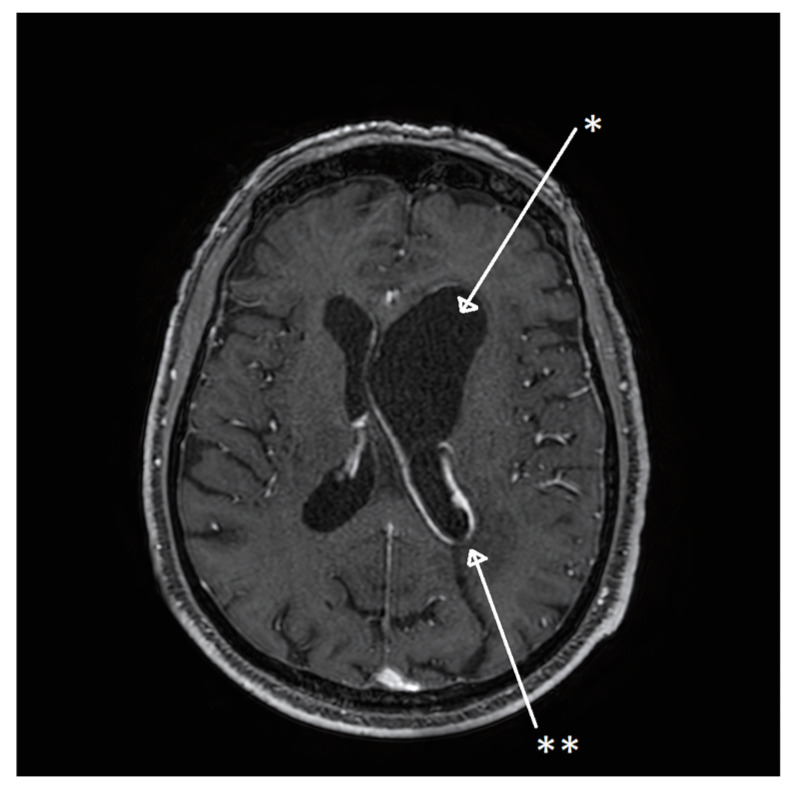
MRI in T1 sequence, with gadolinium contrast enhancement, showing an enlarged left lateral ventricle * and contrast uptake of the excluded ventricle **.

One month after discharge, the patient developed intermittent serotine fever (37.5 °C). At 6 weeks from discharge, the patient presented to the emergency room of our center with altered mental state, gait palsy, aphasia, and strength reduction in the superior right limb. A cerebral MRI with gadolinium revealed an excluded enlarged left lateral ventricle, with a natural hyperintensity in the right frontal lobe presenting in the T2 and FLAIR sequences, as well as in a leptomeningeal contrast enhancement of the walls of the excluded ventricle, suggesting ventriculitis and leptomeningeal inflammation (Figure 2). An emergency external ventricular drain (EVD) was placed in the left lateral ventricle medial wall for intracranial pressure control and CSF examination.

**Figure 3:**
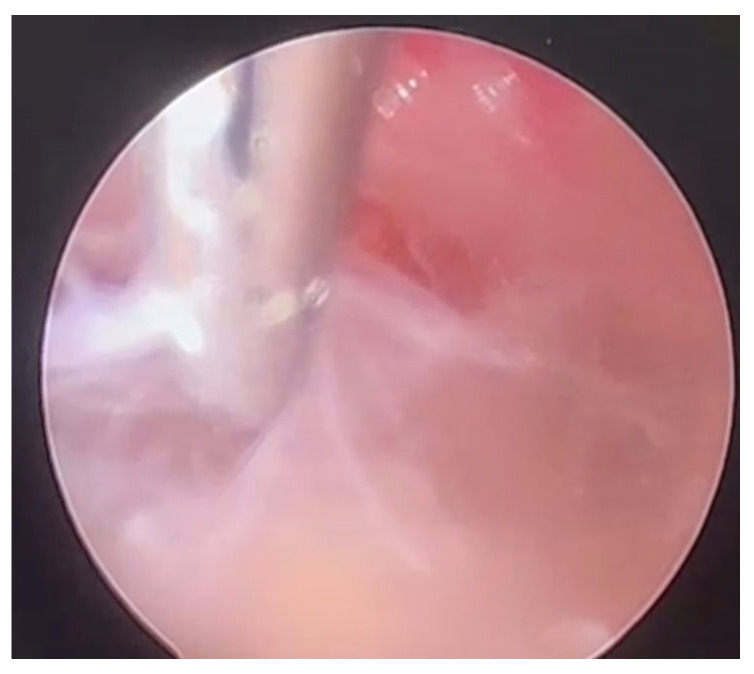
Endoscopic image showing synechiae found in the left excluded ventricle.

The patient began corticosteroid treatment (dexamethasone 32 mg/day) with clinical improvement. CSF analysis showed severe hypoglycorrhachia (19 mg/dL) and hyperproteinorrachia (106 mg/dL) with a normal white blood cell count. All bacterial cultures resulted negative; mycobacterial PCR and an acid-fast bacilli (AFB) smear also resulted negative. To restore CSF circulation within the contralateral ventricles, an endoscopic septostomy through the right frontal horn and an endoscopic exploration of the occipital horn through a left occipital burr hole were performed under neuronavigation. The ependyma appeared to be completely covered by filaments and synechiae (Figure 3), and no communication with the rest of the ventricular system was detectable. A catheter was left in place and connected to an Ommaya reservoir to drain CSF over the next few days. During surgery, a CSF sample was collected directly from the excluded ventricle, and PCR for *Mycobacterium tuberculosis complex* resulted positive, with no molecular resistance.

**Figure 4:**
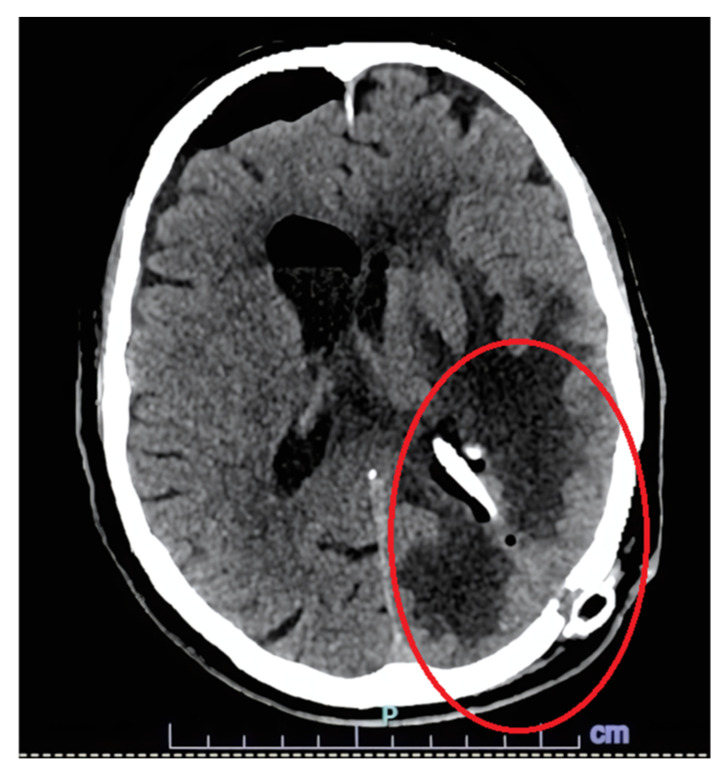
CT scan showing the resolution of hydrocephalus after endoscopic septostomy and placement of Ommaya drain.

Antitubercular therapy was switched from an oral to an intravenous formulation. Linezolid ev 1200 mg/day was added as a fourth antitubercular therapy; this therapy was discontinued after 2 weeks, due to thrombocytopenia, and replaced with levofloxacin 750 mg/day. Dexamethasone was tapered over an 8-week course. A scheduled post-operative CT scan showed resolution of ventricular inflammation and cerebral edema (Figure 4). Before discharge, an LP was repeated, and CSF showed hypoglycorrhachia resolution (45 mg/dL), with a mycobacterial PCR and an AFB smear both negative. Clinically, the patient improved, with some persistent confusion and fatigue, but with fever resolution. The patient was discharged with oral levofloxacin, rifampicin, ethambutol, and isoniazid.

### 2.4. Third Hospital Admission

The patient was admitted again six months after the second hospital discharge due to clinical symptoms of aphasia and confusion. A cerebral CT scan noted hydrocephalus and cysts in the frontal horn of the left lateral ventricle. The Ommaya drain was obstructed and the patient underwent urgent surgery with marsupialization of the frontal horn and cisternostomy. CSF analysis was normal, with negative mycobacterial PCR and cultures. He was discharged with some remaining confusion and behavior disinhibition that resolved in several weeks. He completed 14 months of antitubercular therapy (levofloxacin, rifampicin, ethambutol, and isoniazid). A return to baseline functional status was clinically confirmed at 2 months and maintained at the most recent 10-month follow-up.

## 3. Discussion

As guidelines for BCG immunotherapy-associated CNS infection management are missing, our case report emphasizes the current complexity of the unguided diagnosis and management of CNS BCG infections and provides a comprehensive review of the common management principles available in the literature. Our case is characterized by the microbiological isolation of BCG in the CSF only when collected during surgery, as well as the clinical relapse of symptoms with the development of ventricle obstruction, despite a three-drug antitubercular therapy. A switch to an intravenous four-drug regimen and surgical source control achieved the resolution of symptoms, and clinical cure was sustained up to the 10-month follow-up.

CNS involvement in BCGitis is only anecdotally described, mainly as single case reports. No reviews of CNS BCG infections are currently available. We reviewed all articles indexed in the PubMed, Google Scholar, Embase, ResearchGate, JSTOR, and Science Direct databases from 1988 to March 2025, using the following keywords: Mycobacteria, *Bacillus Calmette-Guerin*, BCG, encephalitis, meningitis, brain abscess, central nervous system infection; all articles not in English or with missing relevant data were excluded. We identified 15 case reports of CNS BCGitis, plus the one described in this article. All cases have been accurately described in Table 1. Eight cases were related to intravesical instillation [4,10,11,12,13,14,15], six to BCG vaccination [16,17,18,19,20] and two were probably related to LP contamination [21]. Patients diagnosed with CNS BCGitis were predominantly male (94%) [4]. As expected, the median patient age associated with BCG intravesical instillation was higher than that for vaccination-associated BCG infection (74 years versus 3 years, *p* < 0.01).

Interestingly, only 31% of the patients identified with our review were reported to be immunosuppressed. This observation invites clinicians to consider BCGitis independently of a patient’s immunocompetence [4,18,20,21]. In the literature, worse outcomes and disseminated disease have been extensively linked with primary immunodeficiencies [23]. More recently, evidence has emerged linking invasive mycobacterial infections to uncommon causes of immunosuppression, such as anti-cytokine antibodies, which are not routinely performed in clinical practice [24,25,26]. The detection of anti-cytokine antibodies may explain disseminated disease in otherwise immunocompetent patients. Since our patient had no immunosuppression history, we tested his convalescent plasma for anti-IL23, anti-IFN-gamma, anti-GM-CSF, and anti-IFN-alpha autoantibodies, which were all negative.

BCG infections are tested with various diagnostic tools, with different diagnostic performance rates. The estimated rates for AFB staining are around 25%, mycobacterial cultures and PCR assays are around 41% each, and overall microbiological diagnoses are around 30% [4]. Histological examination, when possible, reveals granulomatous inflammation in more than 80% of cases [4]. BCG urine isolation cannot be considered diagnostic, since BCG can be isolated in the urine for more than a year after the final instillation of immunotherapy, even in asymptomatic patients [27].

Clinical isolation of BCG in meningitis or ventriculitis is most frequently identified with CSF cultures (n = 15; 94%). Conversely, in patients that developed cerebral abscesses or tuberculomas, BCG isolation was obtained from biopsied cerebral tissue, with CSF cultures often negative. Six out of nine cases of meningitis or ventriculitis showed hypoglycorrhachia, similar to our case [13,16,18,19,21]. In patients that developed cerebral abscesses or tuberculomas, unremarkable or slightly abnormal CSF was usually observed, without any cases of hypoglycorrhachia [10,11,12,17]. In the current patient, as microbiological confirmation of BCG infection was initially missing, a review of the patient’s history and risk factors (including treatment with BCG, the SIADH, and marked hypoglycorrhachia) strengthened our suspicion of a mycobacterial infection [28]. This observation was critical for early BCG-specific empirical antimicrobial therapy, 4 months prior to diagnostic confirmation. In the current patient, a positive PCR for *M. tuberculosis* was achieved only after analyzing the CSF drained during neurosurgery in the excluded ventricle, where we suspected a high bacterial burden.

BCG, being a modified strain of *M. bovis*, is intrinsically resistant to pyrazinamide, cycloserine, and all macrolides, except for clarithromycin [29]. No specific guidelines exist on BCG infection treatment management. Usually, empirical antitubercular therapy in the suspect of systemic BCG infection relies upon the combination of isoniazid, rifampicin, and ethambutol [30,31]. In our review of CNS BCGitis cases, this standard therapy regimen was administered in only 27% of cases [12,16,19], showing a high heterogeneity in therapy regimens with the administration of additional drugs.

Following patient relapse, our therapy regimen included linezolid in combination with standard antitubercular therapy. Linezolid is an antimycobacterial drug suggested for the treatment of MDR- and XDR-resistant TB [32] and has previously been successfully used in non-CNS disseminated BCGitis, but not in CNS BCGitis [33]. We selected linezolid based on its capacity to penetrate the blood–brain barrier and achieve high concentrations in the brain parenchyma [34]. While our patient showed clinical improvement after initiating linezolid, the drug was replaced with levofloxacin after two weeks due to drug-related thrombocytopenia. The choice of fluoroquinolone was driven by evidence that this drug is also effective in achieving a high level in the CNS [35,36]. Inclusion of a fluoroquinolone in the therapy regimen has been reported in other similar cases [4,10,11,13,20].

Notably, we want to emphasize that, even though the BCG strain used for vaccination and intravesical instillation is standardized by the manufacturer, two strains of isolated BCG identified in the literature exhibited unexpected resistance to isoniazid (n = 1) and ethambutol (n = 1). The first patient was treated with rifampicin, ethambutol, and moxifloxacin [10], whereas the second received rifampicin, isoniazid, clofazimine, clarithromycin, ciprofloxacin, and amikacin (for 2 weeks) [20]. These unexpected resistances highlight the importance of conducting antitubercular susceptibility testing for BCG isolates, in case of mycobacterial culture positivity. In our patient’s isolate, no antimicrobial resistance was found.

The use of corticosteroids as adjunctive therapy in BCG infection is controversial. Our review proves that it is administered in less than half of CNS BCGitis cases reported in the literature (6 of 16 patients received corticosteroids). In treating our patient, we decided to follow the indication of tuberculous meningitis guidelines [37,38] and administered high doses of dexamethasone (0.4 mg/kg/day), followed by a taper over 8 weeks.

Despite appropriate oral therapy with three antitubercular drugs, commonly administered in cases of BCG infection (32% in the review by Pèrez et al. [4]), the patient developed obstructive hydrocephalus, requiring endoscopic septostomy and the placement of an Ommaya drain. CSF analysis at the second admission showed a normal white blood cell count (4 cell/mm^3^ vs. 237 cell/mm^3^), but a persistently reduced glycorrhachia, suggesting the persistence of the mycobacteria in the ventricles, responsible for inflammation and ventricle exclusion. We hypothesized that the worsening of symptoms and the formation of the synechiae, observed during surgery, were likely due to inadequate CSF penetration of the three-drug antitubercular therapy initiated during the first admission.

**Figure 5:**
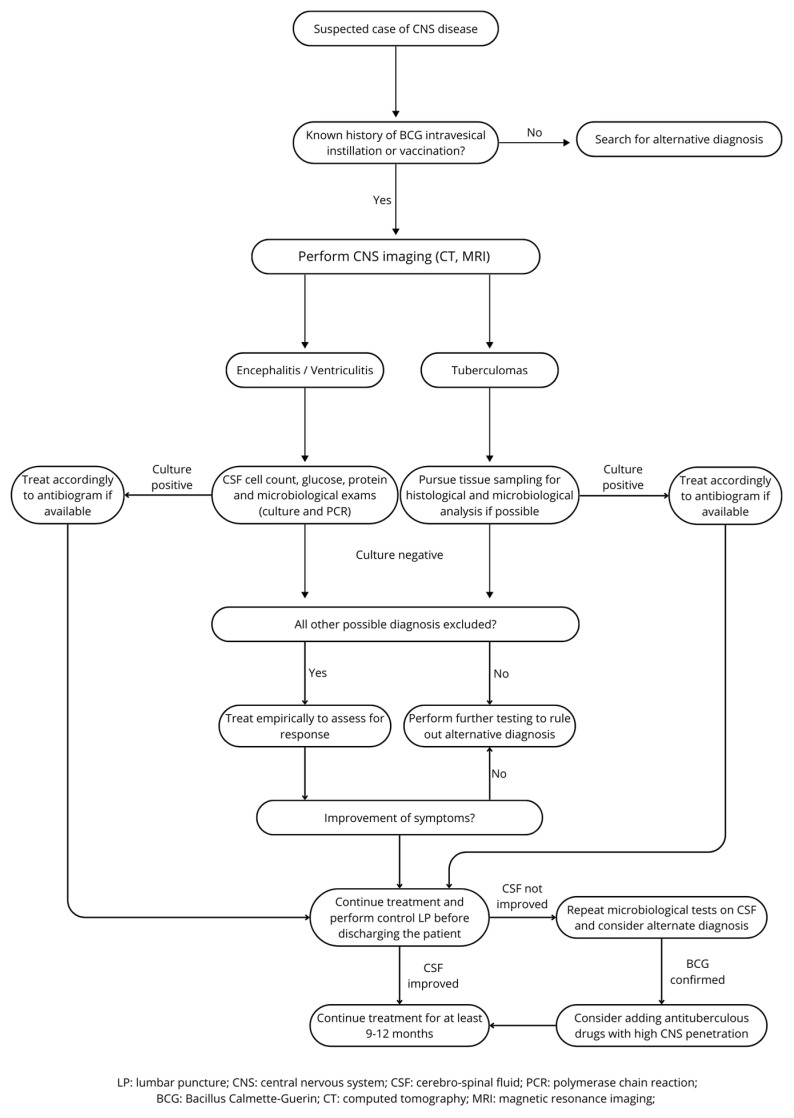
CNS BCGitis management flowchart.

In the absence of clear guidelines, our study group developed a proposed flowchart for optimized CNS BCGitis diagnosis and patient management, see Figure 5.

## 4. Conclusions

CNS BCG infections are multi-faceted diseases. Diagnosis requires a careful history review to identify the patient’s exposure to BCG, exclude alternative diagnoses, and pursue invasive tissue sampling for microbiological investigations. Our patient was the only case described in the literature that had a clinical relapse while on standard antitubercular therapy, with persistent evidence of active infection. A risk–benefit analysis of repeat LP before patient discharge should be investigated as a therapeutic confirmation of CNS BCGitis therapy efficacy. Linezolid may be an alternative choice as a fourth drug in CNS BCGitis, due to optimal CNS penetration, despite the need for strict follow-up for the early identification of possible adverse events.

## Figures and Tables

**Table 1:** Clinical and microbiological characteristics of patients with CNS BCGitis found in literature.

N°	Author [Reference]	Age, Yrs/ Sex	Clinical Presentation	Lesion Site	BCG Infection Type	BCG Isolation Site	CSF Alterations	Therapy	Immuno- Deficient	Adjunctive Corticosteroids	Therapy Duration, Months	Outcome
BCG origin: Intravesical instillations												
1	Golub et al. [10]	73/M	Headaches, dizziness, right hand tremor	Frontal, temporal, and basal ganglia	Tuberculomas	Brain tissue culture	Lymphocytic Pleocytosis and hyperproteinorrachia	RIF + EMB + MOX	No	No	12	SR
2	Parent et al. [11]	89/M	Right upper limb hyposthenia, dysarthria	Multiple left-sided white matter lesions	Vasculitis, endophthalmitis	Brain tissue, humor vitreo	Pleocytosis	INH + RIF + MOX + EMB	No	Yes	N.R.	SR
3	Sheron et al. [12]	74/M	Dizziness, vertigo, gait ataxia	Cerebellum	Abscess	Brain tissue culture	N.R.	INH + RIF + EMB	No	Yes	12	SR
4	Tariq et al. [13]	80/M	Severe ataxia, dysmetria	Nodular leptomeningeal enhancement involving cranial nerves, brainstem, and superior cerebellum	Meningitis	CSF	Lymphocytic Pleocytosis, hypoglycorrhachia, and hyperproteinorrachia	RIF + EMB + MOX	No	Yes	12	Improvement of symptoms; remaining dysmetria
5	Shoskes et al. [14]	67/M	Fever, encephalopathy, respiratory failure	Hyperintense lesions in cortex, subcortical white matter, and brainstem	Miliary infection	Biopsy	N.R.	RIF + INH + EMB + AZM	No	Yes	6	SR
6	Schwartz et al. [15]	88/M	Aphasia	Frontotemporal lesions	Tuberculomas	Tissue biopsy	N.R.	None	No	No	0	Death
7	Pérez-Jacoiste et al. [4]	73/M	N.R.	Inflammation of meninges	Lymphocytic meningitis	Only urine	N.R.	RIF + INH + LEV	Yes	N.R.	6	SR
8	Our case	77/M	Fever, confusion	Right frontal lobe hyperintensity and left lateral ventricle exclusion.	Encephalitis and ventriculitis	CSF	Pleocytosis, hypoglycorrhachia, and hyperproteinorrachia	INH + RIF + EMB + LEV; LIN (2 weeks)	No	Yes	14	SR
BCG origin: Vaccination												
9	Furuichi et al. [16]	1.3/M	gait disturbances	third ventricle	Ventriculitis	CSF and brain tissue	Lymphocytic Pleocytosis, hypoglycorrhachia, and hyperproteinorrachia	INH + RIF + EMB	No	Yes	24	Improvement of symptoms; remaining gait disturbances
10	Sharifi et al. [17]	0.5/M	Poor feeding, nausea, vomiting	Pineal gland	Tuberculoma	Brain tissue culture	Within range	N.R.	No	N.R.	N.R.	SR
11	Van Deutekom et al. [18]	31/M	Fever, neck stiffness, headache	N.A.	Meningitis	CSF	Hyperproteinorrachia and pleocytosis	INH + RIF + PZA	Yes (AIDS)	No	0.3	Death
12	Tardieu et al. [19]	5/M	Fever, rigor	Hyperintensity of subarachnoid space exclusion of fourth ventricle	Ventriculitis	CSF	Pleocytosis, hypoglycorrhachia, and hyperproteinorrachia	INH + RIF + EMB	No	No	18	SR
13	Tardieu et al. [19]	4/M	Fever, rigor	N.A.	Meningitis	CSF	Pleocytosis, hypoglycorrhachia, and hyperproteinorrachia	INH + RIF + EMB	No	No	18	SR
14	Eser et al. [20]	0.25/M	Afebrile seizures	Calcification of the subcortical and periventricular regions	Calcifications, Tuberculomas	Lymph nodes *	Within range	INH + RIF + CAM + CFZ + CIP + AMK (2 mo)	Yes (SCID)	No	12	Microcephaly and persistence of axial hypotonia
BCG origin: Unknown (LP contamination?)												
15	Stone et al. [21]	3/F	Headache	N.A.	Meningitis	CSF	Pleocytosis	INH + RIF + ETI + STP	Yes (ALL in active chemotherapy *)	No	12	SR
16	Stone et al. [21]	5/M	Headache, fever, weight loss	N.A.	Meningitis	CSF	Pleocytosis, hypoglycorrhachia, and hyperproteinorrachia	INH + RIF + STP + ETI	Yes (ALL in active chemotherapy *)	No	12	SR

BCG: *Bacillus Calmette–Guerin*; CSF: cerebrospinal fluid; RIF: rifampicin; EMB: ethambutol; MOX: moxifloxacin; CIP: ciprofloxacin; AMK: amikacin; CAM: clarithromycin; CFZ: clofazimine; INH: isoniazid; PZA: pyrazinamide; ETI: ethionamide; STP: streptomycin; LEV: levofloxacin; AZM: azithromycin; LIN: linezolid; N.R.: not reported; N.A.: not applicable; SR: symptom resolution; ALL: acute lymphoblastic leukemia; SCID: severe combined immunodeficiency. * see comment in the main text.

## Data Availability

The data presented in this study are available on request from the corresponding author, due to privacy restrictions.

## Abbreviations

The following abbreviations are used in this manuscript:

BCG	*Bacillus Calmette–Guerin*
MRI	Magnetic resonance imaging
CSF	Cerebrospinal fluid
NMIBC	Non-muscle-invasive bladder cancer
CNS	Central nervous system
EEG	Electroencephalography
LP	Lumbar puncture
CT	Computed tomography
SIADH	Inappropriate antidiuretic hormone secretion
EVD	External ventricular drain
AFB	Acid-fast bacilli
AIDS	Acquired immunodeficiency syndrome
RIF	Rifampicin
EMB	Ethambutol
MOX	Moxifloxacin
INH	Isoniazid
PZA	Pyrazinamide
ETI	Ethionamide
STP	Streptomycin
LEV	Levofloxacin
ALL	Acute lymphoblastic leukemia
SCID	Severe combined immunodeficiency
CFZ	Clofazimine
CAM	Clarithromycin
CIP	Ciprofloxacin
N.R.	Not reported
AMK	Amikacin
N.A.	Not applicable
SR	Symptom resolution
